# The Neurostimulationist will see you now: prescribing direct electrical stimulation therapies for the human brain in epilepsy and beyond

**DOI:** 10.3389/fnhum.2024.1439541

**Published:** 2024-09-04

**Authors:** Peter N. Hadar, Rina Zelmann, Pariya Salami, Sydney S. Cash, Angelique C. Paulk

**Affiliations:** ^1^Department of Neurology, Massachusetts General Hospital, Harvard Medical School, Boston, MA, United States; ^2^Center for Neurotechnology and Neurorecovery, Department of Neurology, Massachusetts General Hospital, Boston, MA, United States

**Keywords:** direct electrical stimulation, intracranial, human, neural activity, epilepsy

## Abstract

As the pace of research in implantable neurotechnology increases, it is important to take a step back and see if the promise lives up to our intentions. While direct electrical stimulation applied intracranially has been used for the treatment of various neurological disorders, such as Parkinson’s, epilepsy, clinical depression, and Obsessive-compulsive disorder, the effectiveness can be highly variable. One perspective is that the inability to consistently treat these neurological disorders in a standardized way is due to multiple, interlaced factors, including stimulation parameters, location, and differences in underlying network connectivity, leading to a trial-and-error stimulation approach in the clinic. An alternate view, based on a growing knowledge from neural data, is that variability in this input (stimulation) and output (brain response) relationship may be more predictable and amenable to standardization, personalization, and, ultimately, therapeutic implementation. In this review, we assert that the future of human brain neurostimulation, via direct electrical stimulation, rests on deploying standardized, constrained models for easier clinical implementation and informed by intracranial data sets, such that diverse, individualized therapeutic parameters can efficiently produce similar, robust, positive outcomes for many patients closer to a prescriptive model. We address the pathway needed to arrive at this future by addressing three questions, namely: (1) why aren’t we already at this prescriptive future?; (2) how do we get there?; (3) how far are we from this Neurostimulationist prescriptive future? We first posit that there are limited and predictable ways, constrained by underlying networks, for direct electrical stimulation to induce changes in the brain based on past literature. We then address how identifying underlying individual structural and functional brain connectivity which shape these standard responses enable targeted and personalized neuromodulation, bolstered through large-scale efforts, including machine learning techniques, to map and reverse engineer these input–output relationships to produce a good outcome and better identify underlying mechanisms. This understanding will not only be a major advance in enabling intelligent and informed design of neuromodulatory therapeutic tools for a wide variety of neurological diseases, but a shift in how we can predictably, and therapeutically, prescribe stimulation treatments the human brain.

## 1 A future neuromodulation clinic visit

This review focuses primarily on epilepsy because many of the recent innovations in neuromodulation and intracranial neural activity have come from this field. To illustrate the broader applicability of the reviewed approaches we start by presenting a vignette of a possible future neuromodulation clinic visit for another disorder, clinical depression. Based on the advances in epilepsy neurostimulation, we hope that implanted, responsive neurostimulation may one day become the standard of care for a wider variety of neurologic and psychiatric disorders.

**Figure 1:**
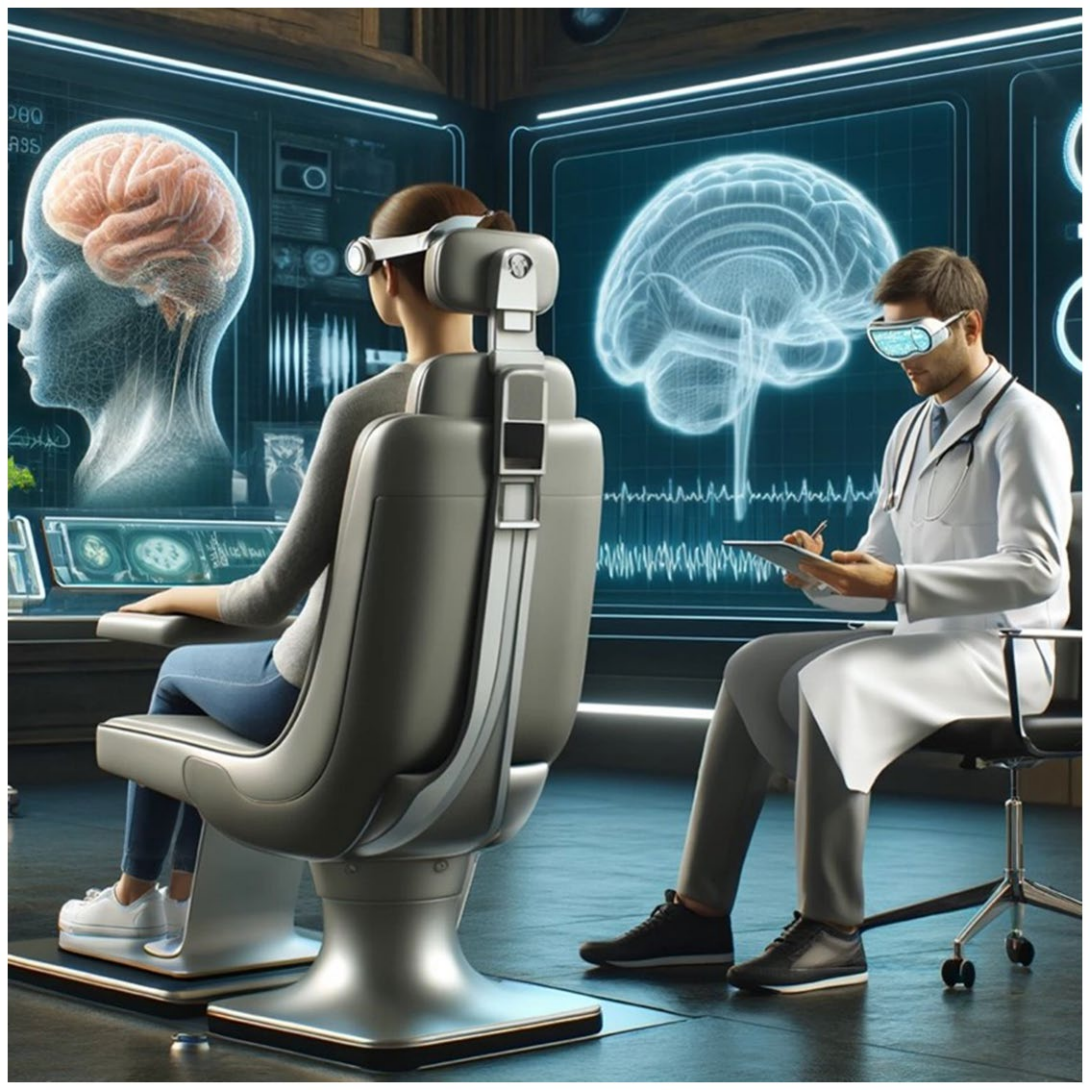
AI-rendered neurostimulation future. A Dall-E rendering of what a future clinic visit could look like when prompted, “Draw me a picture of a doctor’s office in 100 years, where a neurologist will be sitting with AR Goggles and a tablet, and a patient will be sitting on the chair. There will be a screen in the background that has a 3D picture of the patient’s brain and their brainwaves.”

July 1, 2054. Dr. Smith, a neuromodulation-trained neurologist, is seeing the fifth patient of the day with an implanted, closed-loop neurostimulator for depression (Figure 1). After consistent EEG findings were determined to be linked to depressive symptoms in the 2030s, a closed-loop neurostimulator for depression was developed in the 2040s; neurostimulationists have been tracking various depressive symptoms and discovered clear categories that respond to specific neuromodulation parameters. By 2054 this approach is standard of practice. A robotic surgery was performed approximately a month before, and now the neurostimulator has been recording the patient’s brain activity for the last few weeks. Before the patient comes in, Dr. Smith checks the alert at the top right of his visors’ screen which indicates that, based on the last month of electrocorticography data collected, the patient falls into the “Depression-Severe-E” type with markers mapping to identified Research Domain Criteria matrices. The notification asks if he wants to load up the standardized initial neurostimulation parameters for this diagnosis, and Dr. Smith indicates yes. Informed by brain network imaging with AR glasses, Dr. Smith adjusts the parameters and the initial variable adjusting algorithm that can adapt to the initial phase of treatment. After Dr. Smith brings in the patient, they talk about how she is feeling, and she states that she is still feeling depressed. Dr. Smith implements the “Depression-Severe-E” parameter set, and the patient notes that she felt slightly brighter. Pleased with the initial response and that the system is detecting the right signals for closed loop adaptation, Dr. Smith said the full effect would take about 6–8 weeks to manifest, and they schedule a follow-up appointment in 2 months virtually, with a plan to modify the stimulation remotely at the next visit.

## 2 Introduction

While this vision of a future where neurostimulation can be prescribed and customized for individual patients can appear far-fetched (Figure 1), the research currently being conducted, and which will be discussed in this review, is laying the groundwork to turn this neurotherapeutic stimulation approach into a reality. Of note, for the purposes of this article, we will be focusing primarily on epilepsy because many of the recent advances in neurostimulation and direct intracranial data have come out of this space. However, there are three major questions we can ask to get to this space: (1) *Why aren’t we there now?* (2) *How do we get there?* and (3) *How far are we from a Neurostimulationist prescription vision of personalized, closed-loop neurostimulation therapy?*

To address the first question, a major reason the neuromodulation field has yet to reach this future ideal in large part is because the stimulation delivery system and approach require customized placement of intracranial electrodes to targets that vary in size and shape from person to person combined with an almost infinite stimulation parameter space (see below). Chronically and semi-chronically implanted electrodes used to apply direct electrical stimulation (DES), including through responsive neurostimulation system (RNS) or deep brain stimulation (DBS) devices, involve voltage or current applied through intracranial leads (Herrington et al., 2016). Chronically implanted DES has become an important diagnostic and therapeutic tool for neuropsychiatric diseases ranging from Parkinson’s disease to obsessive compulsive disorder (OCD) to epilepsy and depression (Berger and Ojemann, 1992; Rizzone et al., 2001; Mayberg et al., 2005; Bronstein et al., 2011; Rolston et al., 2011; Bourne et al., 2012; Lozano and Lipsman, 2013; Revell, 2015; Herrington et al., 2016; Lozano et al., 2019; Goodman et al., 2020; Nair et al., 2020; Sheth et al., 2021; Scangos et al., 2021a; Shlobin et al., 2022; Vissani et al., 2023), with approaches recently expanding to tackling stroke and traumatic brain injury (Kundu et al., 2018; Baker et al., 2023; Schiff et al., 2023). Semichronic implantations can involve DES via intracranial leads during ongoing monitoring for seizure networks up to 29 days and comprise many of the currently available data sets demonstrating how the brain responds to stimulation (Trebaul et al., 2018; Basu et al., 2019; Mohan et al., 2020; Sheth et al., 2021; Scangos et al., 2021a; Paulk et al., 2022; Zelmann et al., 2023). Alternately, the chronically implanted DES devices are starting to be considered to not only neuromodulate underlying brain activity, but also serve as a neurotherapeutic tool to restore broader function.

As a case in point, this review article will use neuromodulation in patients with epilepsy as an example of getting closer to achieving this vision while addressing complex factors since the field of epilepsy neuromodulation has some of the more advanced neurostimulators involving varying stimulation timing and patterns with closed loop adaptation (Bergey et al., 2015; Lee et al., 2015; Nair et al., 2020; Jarosiewicz and Morrell, 2021; Anderson et al., 2024), adding the complication of variability to the complexity of the stimulation input and the possible output. This point is central to the answer of “*Why aren’t we there now?*”, namely, the problem is that stimulation parameters, underlying brain regions, and effects of state can alter stimulation effectiveness. To address this challenge, recent advances in neurostimulation have progressed beyond individual detection or treatment and sought to combine them in an algorithmic approach. The NeuroPace RNS device, used for epilepsy, allows for the implementation of algorithmically-based detection of epileptiform features in neural activity which can be partnered with a provider-determined neurostimulation treatment to personalize the neurostimulation to the patient’s seizure activity (Table 1; Bergey et al., 2015; Lee et al., 2015; Jarosiewicz and Morrell, 2021). Long-term use of this device has pointed to a possibility that the stimulation is modifying the underlying network (Kokkinos et al., 2019; Nair et al., 2020; Khambhati et al., 2021; Charlebois et al., 2022; Anderson et al., 2024), possibly related to this idea of a “neurotherapeutic” effect of stimulation.

**Table 1:** RNS now and in the future.

RNS for epilepsy	Current reality	Ideal future
ECOG recordings	Few short epochs of recordings per day	Live, 24/7 ECOG Recordings
Upload	Patient uploads	Continuously uploaded to cloud
Programming	In-person visit	Remote programming
Alerts	None	Physician alerted for increased seizure activity
Programming parameters	Largely trial and error with increasing charge density	Machine Learning/Connectivity-based and Individualized Parameters automatically adapt to changing ECOG signal

Current recording, stimulation, and interface issues with the responsive neurostimulator systems with a future ideal hope of onboard models and neural data to better inform clinical decisions. ECoG, electrocorticography recordings.

Unfortunately, and related to the first main question of “*Why aren’t we there now?*” in this Neurostimulationist prescriptive vision, even with these advanced, responsive devices that can be customized to a patient’s neural activity, many patients still do not receive a therapeutic benefit from neurostimulation (Rolston et al., 2011; Dougherty et al., 2015; Crowell et al., 2019; Scangos et al., 2021c; Shlobin et al., 2022). A significant factor is thought to be the trial-and-error approach needed to stimulation setting adjustments (e.g., responsive neurostimulator or RNS for epilepsy, deep brain stimulation or DBS for Parkinson’s disease; Bronstein et al., 2011; Eisinger et al., 2019; Kokkinos et al., 2019; Nair et al., 2020), which can often lead to numerous failed trials and poor outcomes (Harvey and Winstein, 2009; Hardenacke et al., 2013; Dougherty et al., 2015; Kuhn et al., 2015; Crowell et al., 2019). Since it is still unclear how or why some neurostimulation parameters are effective, it is often difficult to determine which programmed neurostimulation parameter set is ideal for each patient at a given stage of their treatment (Cagnan et al., 2017, 2019; Herrington et al., 2016; Widge et al., 2017, 2018; Kokkinos et al., 2019). Further, the original idea of RNS for epilepsy is to use stimulation to stop the seizure when it is detected, but a recent paper has highlighted the possibility that any intermittent stimulation in between seizures could be enough to modulate the network and reduce seizure load (Anderson et al., 2024).

Another challenge is that the implantable chronic RNS devices can only record and stimulate in short snippets of time and require patient visits at the doctor’s office, and active participation of the patient with no alerts, giving the provider an incomplete picture of seizure activity and effects of stimulation (Table 1). An ideal arrangement would be to enable continuous recording, alert systems for the patient and provider, and, importantly, identify optimal stimulation parameters for that participant and adjust based on ongoing neural network changes. To get to this point, though, the next major question to address is “*How do we get to that Neurostimulationist vision of personalized closed loop therapy?*”

That path involves identifying common “standard” stimulation strategies for neurotherapeutic modulation which can be modified per participant, informed by models shaped by multi-participant data sets (including more than 100 individuals). Based on a growing body of literature, we propose that there is a consistency in neural responses with less variability than previously thought (Trebaul et al., 2018; Basu et al., 2019; Mohan et al., 2020; Paulk et al., 2022). Some degree of consistency could allow for standardization, personalization, and implementation of novel neurostimulation approaches. Further, the power of machine learning (Thangavel et al., 2021) as well as improving connectivity measures using noninvasive imaging and other tools have revolutionized the DBS field for movement disorders and could be critical in identifying the ideal targets per participant (Horn et al., 2017; Riva-Posse et al., 2018; Horn and Fox, 2020; Middlebrooks et al., 2021). In the face of consistent response dynamics and major advances in connectomics and machine learning, we are closer to achieving a therapeutic neurostimulation approach based on standards instead of trial-and-error and our perspective piece aims to propose a way to get there (Bergey et al., 2015; Nair et al., 2020).

## 3 Why aren’t we there now? Stimulation input–output complexity and the need for identifying standardized stimulation responses across individuals

### 3.1 Are there too many possible inputs and outputs, or is there a pattern?

**Figure 2:**
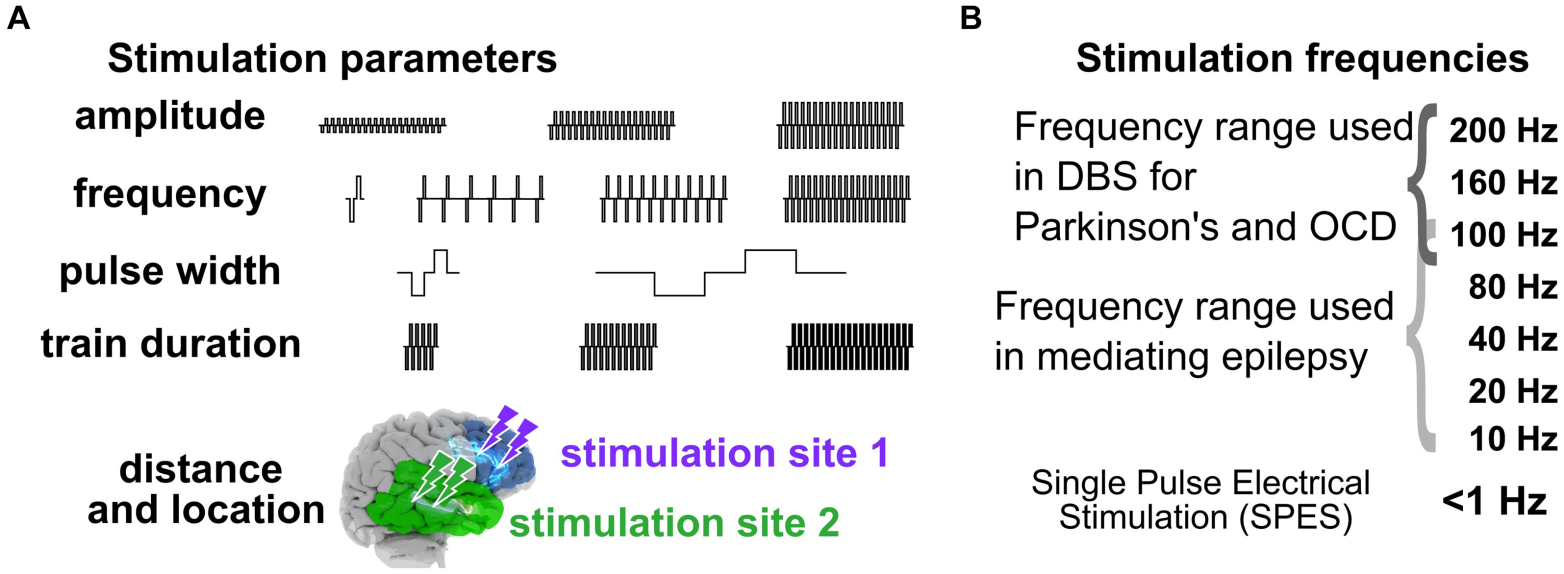
Stimulation parameters to induce neural responses. **(A)** Varying stimulation parameters used to induce behavioral and physiological changes. **(B)** Stimulation frequencies. The combination of stimulation parameters can therefore represent a massive and daunting space when stimulating intracranially to get a targeted response.

In addressing the first question of “*Why aren’t we there now?*,” the crux of the challenge of moving from trial-and-error approaches to a standardized, predictable prescriptive approach with neuromodulation, particularly in the epilepsy space, is the seemingly “infinite” number of stimulation pattern combinations and brain locations, underlying brain network features, and brain states (Figure 2). On one hand we have to deal with variability in stimulation parameters including frequency, amplitude, waveform duration and more, and on the other hand, we have variability in electrode placement relative to targeted networks and brain regions (Figure 2; Mikulan et al., 2020; Crocker et al., 2021; Russo et al., 2021; Parmigiani et al., 2022; Paulk et al., 2022). For instance, the Neuropace RNS device provides 12,672,000 total number of combinations of stimulation parameters (frequency X amplitude steps X burst duration steps X pulse duration steps) alone.

**Figure 3:**
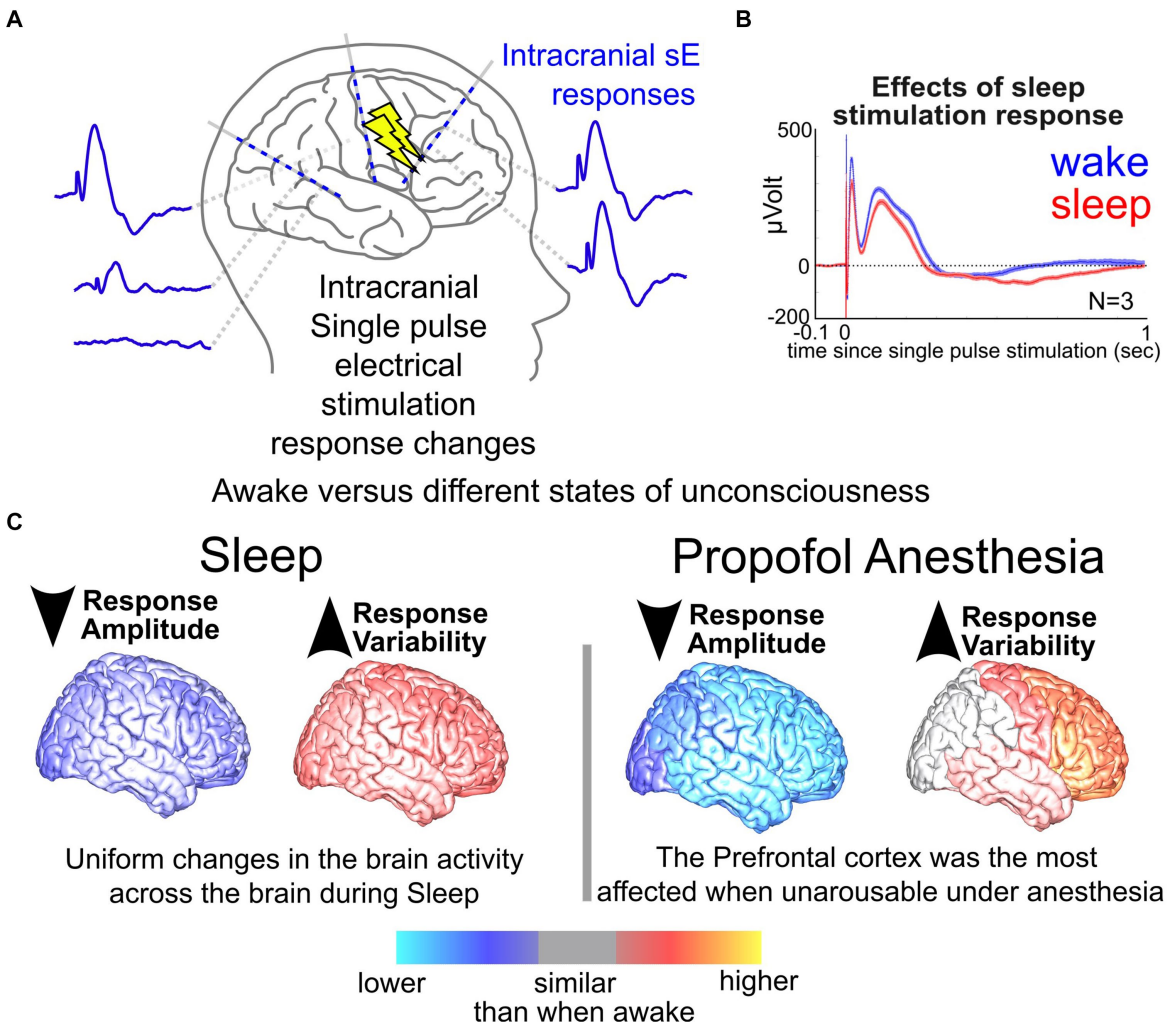
Neural responses in sleep vs. wake. **(A)** Illustration of stimulation responses via stereo-EEG (sEEG) electrodes implanted in the brain. **(B)** Changes in a response to single pulse electrical stimulation (SPES) dorsolateral prefrontal cortex site during wake vs. sleep. **(C)** Measured changes in brain responses to SPES with stimulation at different sites of the brain during sleep and anesthesia compared to when the participants were awake (Zelmann et al., 2023).

In addition to neurostimulation inputs and brain response outputs, the timing of stimulation can have a modulatory effect. Our group, for example, has previously shown that the brain state (e.g., sleep or awake) alters both stimulation responses and network connectivity (Pigorini et al., 2015; Stiso et al., 2019; Salami et al., 2020, 2022; Zelmann et al., 2020, 2023; Russo et al., 2021); the latter, when mapped, could improve stimulation efficacy (Sheth et al., 2021; Scangos et al., 2021a, 2021c; Bahners et al., 2022; Alagapan et al., 2023). The neural impact and behavioral effectiveness of stimulation depend critically on the current physiological and behavioral state of the individual (Jiruska et al., 2010; Cagnan et al., 2017; Hoang et al., 2017; Ezzyat et al., 2018; Solomon et al., 2018). However, little work has been done systematically exploring the potential role overall brain state plays in the effectiveness of stimulation (Kawaguchi et al., 1996; Peyrache et al., 2012; Usami et al., 2015, 2017, 2018; Zelmann et al., 2023). In a previous study, we explored the differences in responses to single pulses during sleep versus wake (*N* = 17 wake, *N* = 13 asleep, Figure 3) and found that responses to single pulses during wake were overall smaller across participants (Zelmann et al., 2023). Further, stimulation-induced network connectivity selectively changed across the brain, with an increase in response variability during sleep (Zelmann et al., 2023). Factoring in the stimulation parameter space, effects of brain state, and underlying network connectivity of the individual, one could imagine just how daunting and possibly unpredictable the input (stimulation) and output (brain response) relationships could appear.

One answer to the next question, “*How do we get to that Neurostimulationist vision of personalized closed loop therapy?*,” lies in the progress of data sharing initiatives, standardization of data sets, even intracranial brain recording data sets. There has been a substantial expansion of the interpretable and useable data to better understand how the human brain responds to intracranial stimulation as well as enable cross-site comparisons across a larger population of individuals (Holdgraf et al., 2019; Mercier et al., 2022; Duncan et al., 2023). By extension, one issue could be that this expansion, and even democratization, of the intracranial stimulation space results in a notable increase in the modifiable variables to induce changes in activity. However, we would argue that the opposite may be true: By taking a wider view of these large data sets gathered and shared, response patterns could be emerging across individuals and stimulation parameters that may make it possible to standardize neuromodulatory approaches.

### 3.2 The commonality of input–output relationships across individuals is informed by a mechanistic understanding of stimulation effects

**Figure 4:**
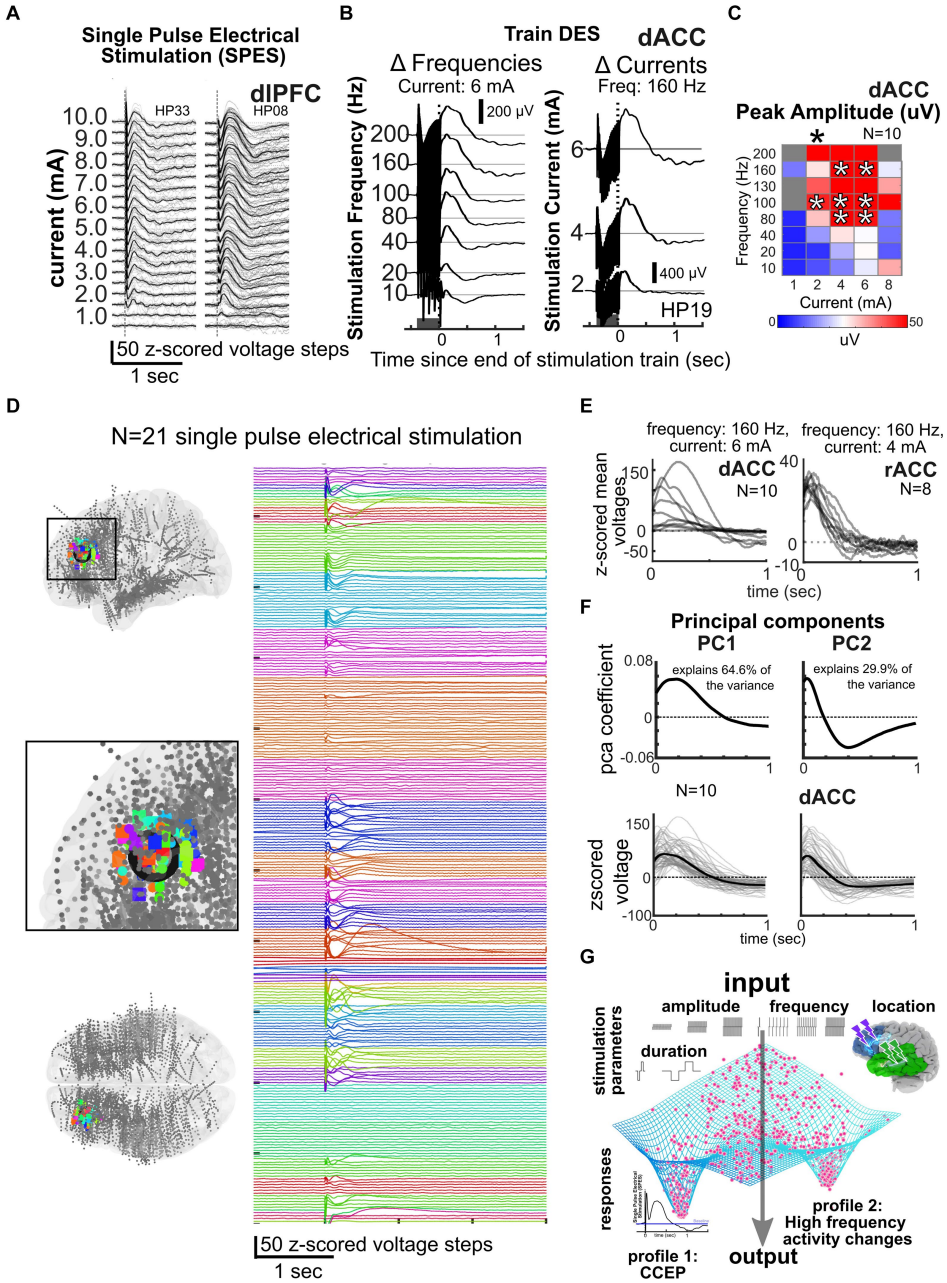
Data reduction and predicting responses across participants and frequencies. **(A–B)** Responses to single pulse stimulation **(A)** or trains of stimulation (dark grey bar, **B**) or for different frequencies and currents (*N* = 3). **(C)** Current × frequency map of voltage responses across individuals for a voltage response (*N* = 10). **(D)** Local stimulation responses across 21 participants with stimulation at the color coded sites shown in the MNI-mapped electrode locations on the left in the dorsolateral prefrontal cortex, illustrating the consistency of responses across individuals and stimulation sites (Paulk et al., 2022). **(E)** Example dorsal anterior cingulate (dACC) and rostral anterior cingulate (rACC) stimulation responses after a train of stimulation to specific current and frequency levels across multiple individuals. **(F)** Cingulate and amygdala responses decomposed into principal components, with 94.5% of the variance of local, nearby voltage responses (bottom) explained by principal components 1 and 2. Linear models (LM) incorporating current and frequency with principal component (PC) coefficients can be used to predict responses (bottom; Basu et al., 2019). **(G)** General framework of the idea of an output attractor state type funneling of brain responses such as the CCEP or high frequency activity changes in spite of a wide variety of input stimulation parameters.

One major reason we continue to ask “*Why aren’t we there now?*” is that we have a limited understanding of the mechanisms of how stimulation alters human brain activity, though this is changing, particularly in the past few years. Overall, primary stimulation parameters most often adjusted during therapies and in existing large data sets include electrode location, distance of the recording site to the stimulating electrode, current amplitude, pulse width, stimulation train duration, and stimulus frequency (Basu et al., 2019; Mikulan et al., 2020; Mohan et al., 2020; Crocker et al., 2021; Parmigiani et al., 2022; Paulk et al., 2022). We posit that the factor with the largest impact on stimulation responses is frequency, which can range from single pulse electrical stimulation (SPES) occurring every 1–5 s to trains of stimulation up to 200 Hz (Figures 1, 4). SPES induces highly consistent local voltage response profiles per brain region (Matsuzaki et al., 2013; Keller et al., 2014a, 2014b; Matsumoto et al., 2017; Yukihiro et al., 2017; Trebaul et al., 2018; Mikulan et al., 2020; Crocker et al., 2021; Parmigiani et al., 2022) and is used to map pathologies such as epilepsy (Boulogne et al., 2016; Matsumoto et al., 2017; Hebbink et al., 2020). Trains not only induce different neural dynamics compared to SPES (López, 2001; Brocker and Grill, 2013; Saito et al., 2015; Boulogne et al., 2016; Basu et al., 2019; Yih et al., 2019; Kundu et al., 2020; Parmigiani et al., 2022; Paulk et al., 2022), but form the basis for all therapeutically delivered stimulation (Garcia et al., 2005; Laxton et al., 2013; Mahlknecht et al., 2015; Chiken and Nambu, 2016; Herrington et al., 2016; Eisinger et al., 2019). High frequency train stimulation (above 100 Hz) has been shown to be therapeutic in Parkinson’s disease and OCD (Rizzone et al., 2001; Bronstein et al., 2011; Bourne et al., 2012; Provenza et al., 2019; Goodman et al., 2020; Grover et al., 2021; Sheth et al., 2021), but lower frequencies (<100 Hz) alter neural activity therapeutically in epilepsy (Lee et al., 2015; Kokkinos et al., 2019; Nair et al., 2020) or memory consolidation (Ezzyat et al., 2017, 2018; Titiz et al., 2017; Mankin and Fried, 2020).

Fortunately, we are at a pivotal point as our group and others have been methodically mapping the stimulation input-response output space and, importantly, have been sharing these data as part of Open Data initiatives (Solomon et al., 2018, 2021; Basu et al., 2019; Holdgraf et al., 2019; Kundu et al., 2020; Mohan et al., 2020; Qiao et al., 2020; Crocker et al., 2021; Sheth et al., 2021; Adkinson et al., 2022; Parmigiani et al., 2022; Paulk et al., 2022; Zelmann et al., 2023). Multiple research centers are finding consistent and identifiable repertoires of stimulation responses across participants and patients per brain region and combination of these parameters (Matsuzaki et al., 2013; Keller et al., 2014a, 2014b; Matsumoto et al., 2017; Yukihiro et al., 2017; Solomon et al., 2018, 2021; Trebaul et al., 2018; Basu et al., 2019; Kundu et al., 2020; Mohan et al., 2020; Crocker et al., 2021; Huang et al., 2022; Paulk et al., 2022). We propose that a pattern is emerging across these efforts which is, namely, that multiple combinations of parameters can achieve a diverse, though limited repertoire of physiological responses. Indeed, the human brain could be acting to convert stimulation input into a subset of replicable responses (Basu et al., 2019; Huang et al., 2022), which would enable chart a path to reliable treatments for a wide range of proposed neurological pathologies (Mayberg et al., 2005; Lozano and Lipsman, 2013; Widge et al., 2017; Trebaul et al., 2018; Lozano et al., 2019; Mankin and Fried, 2020). This is likely why we found the same or similar waveform components across humans and even non-human primates with correlation values >0.75 between voltage responses between individuals per brain region (Grill et al., 2004; Grill and Wei, 2009; Kent et al., 2015; Kanno et al., 2018; Yih et al., 2019).

To understand the patterns of these stimulation responses, we have to understand the mechanisms underlying stimulation to the human brain (Grill et al., 2004; Brocker and Grill, 2013; Herrington et al., 2016; Russo et al., 2024). For instance, a number of studies show neural responses appear to involve slow voltage curves involving multiple peaks (N1, N2) thought to be associated with excitatory and inhibitory dynamics, with response variations involving changing amplitude or time courses (Figure 4; Matsuzaki et al., 2013; Keller et al., 2014a, 2014b; Kunieda et al., 2015; Matsumoto et al., 2017; Yukihiro et al., 2017; Trebaul et al., 2018; Basu et al., 2019; Mikulan et al., 2020; Crocker et al., 2021; Parmigiani et al., 2022; Zelmann et al., 2023). These responses could also be due to thalamic feedback which could be mapped using imaging and other tools (Russo et al., 2024). Not surprisingly, these evoked responses can saturate with increasing stimulation amplitude (current) as the underlying tissue could be either saturating in its responses or the inhibitory network is engaged to suppress overexcitation (Parmigiani et al., 2022; Paulk et al., 2022; Russo et al., 2024).

Another reported stimulation response type across studies involves changes in high gamma (30–200 Hz range) oscillatory power (Grill et al., 2004; Mohan et al., 2020; Scangos et al., 2021c, 2021a; Alagapan et al., 2023). These response repertoires can occur with the same ranges of stimulation parameters but with a high level of consistency within and across participants. Specifically, the trains of 100–200 Hz pulses inducing the largest voltage responses (and a corresponding decrease in high frequency activity, HFA, 30–100 Hz) for a range of current levels (Basu et al., 2019; Mohan et al., 2020) are so consistent that we observe nonlinear “hot spots” of responses in the current x frequency matrices across individuals (Figure 4; Mohan et al., 2020). Notably, these hotspots are not small, localized points but, instead, show that there are similar responses to a range of similar frequencies, currents, and stimulation patterns across individuals and per brain region. These results could also be highlighting an important point about the brain: beyond certain frequencies, currents, or other ranges, either saturating responses or the inhibitory network could be engaged to limit local activity, effectively a temporary “lesion” of activity in that region or thalamocortical networks are engaged that can lead to predictable responses (Grill et al., 2004; Russo et al., 2024). Either way, ultimately, we are positing that there are a finite number of waveforms and amplitudes and frequency responses available across the full range of stimulation parameter combinations driven by features of underlying neural tissue.

### 3.3 Proposed intervention: limiting the parameter space guided by large data sets

An alternative, grounded on recent studies and shared dataset, is that a combination of the limited but diverse repertoire of responses could explain the complexity observed in different contexts or brain states. Further analysis is needed to test this central hypothesis and the generalizability of these findings, especially whether they can predict stimulation parameters that induce brain region specific stimulation-induced neural biomarkers identified in the literature as being clinically relevant (Lee et al., 2015; Miocinovic et al., 2018; Provenza et al., 2019; Nair et al., 2020; Basu et al., 2021; Middlebrooks et al., 2021; Provenza et al., 2021; Sheth et al., 2021; Scangos et al., 2021a, 2021c; Bahners et al., 2022; Alagapan et al., 2023; Merk et al., 2023). However, even at a first pass, we propose that we can generate maps or atlases per stimulated sites in different regions (e.g., cingulate vs. lateral frontal lobe) by taking into account multidimensional stimulation parameters and demonstrate common response properties across participants (Figure 4). To highlight this point, in a data set of *N* = 52 participants with *n* = 750 possible stimulation sites and *n* = 6,500 recording sites mapped to a common MNI space, we have found similar response patterns to SPES across individuals (indicated by different colors) when examining nearby contacts to the stimulation sites (in the dorsolateral prefrontal cortex) as well as in the contralateral homologous region (Figure 4). Indeed, this type of large data set mapping across individuals will, we propose, be key to answering the next question of “*How do we get to that Neurostimulationist vision of personalized closed loop therapy?*” by reducing the stimulation space to a subsampled set of critical parameters for modulating human brain activity.

Further support for this idea of clustered response profiles in the neural activity comes from comparing the evoked potentials in response to local stimulation across individuals, thus allowing us to predict responses to combinations of stimulation parameters. In a smaller initial data set of awake participants (N = 18 with stimulation in the dorsal anterior cingulate cortex, dACC, and the rostral anterior cingulate cortex, rACC), we have successfully used principal components analysis (PCA) dimension reduction techniques on the voltage time series combined with generalized linear models (GLM) to predict voltage responses from stimulation parameters (Figure 4; Basu et al., 2019). Not only did the first two principal components generated from voltage responses explain 94.5% of the variance across individuals, but also PCA combined with GLM allowed us to predict stimulation responses to different frequency and current steps. Emphasizing how consistent this pattern is across individuals, we could also predict responses in different patient participants from a subset of other participants using the same underlying GLMs (Basu et al., 2019; Paulk et al., 2022). Further, responses cluster across the 52 total participants using PCA and k-means clustering tools.

Of course, this work would need to be expanded upon using GLM, PCA, and clustering analyses to identify optimal minimal clusters in a significantly larger data set (N > 100 s of individuals; Basu et al., 2019; Mohan et al., 2020; Paulk et al., 2022; Zelmann et al., 2023) with more variation due to varying states (sleep vs. wake; Zelmann et al., 2023) to demonstrate that, not only do responses cluster to a limited repertoire of responses, but also these responses are predictable across ranges of stimulation parameters, brain states, and across individuals. However, the standardized mapping, common predictability of the evoked responses, and overall nonlinear hot spot response profiles emphasize a central point: We do not need to sample every frequency x current x pulse duration step per widespread brain region but could use a general atlas to navigate, and narrow, the massive parameter space.

## 4 How do we get there? Personalization through machine learning and connectivity analyses

While standardizing our neurostimulation approaches and limiting the search space are important steps, the promise of neuromodulation treatment will need to incorporate personalized features of the individual, characterized by personalized neuroanatomical (structural), functional (correlated activity), and effective (directional) connectivity of each individual brain (Friston, 2011; Donos et al., 2016; Horn et al., 2017; Crocker et al., 2021; Charlebois et al., 2022). Brains are wired differently between individuals, especially in the patient populations engaged in this neuromodulatory space (such as those with epilepsy) compared to the brains of healthy controls (Charlebois et al., 2022). However, recent identification of clinically relevant neural biomarkers in the neural signals combined with machine learning (ML) and connectivity analyses on brain data could be the keystone leading to personalized neurostimulation maps and could even inform clinical management. Therefore, the development of this multimodal and predictive approach could be critical to answering the second question:” “*How do we get to that Neurostimulationist vision of personalized closed loop therapy?*”

### 4.1 Brain network connectivity is key for personalized neurostimulation treatment

#### 4.1.1 Stimulation responses are constrained by network connectivity and neural architecture

**Figure 5:**
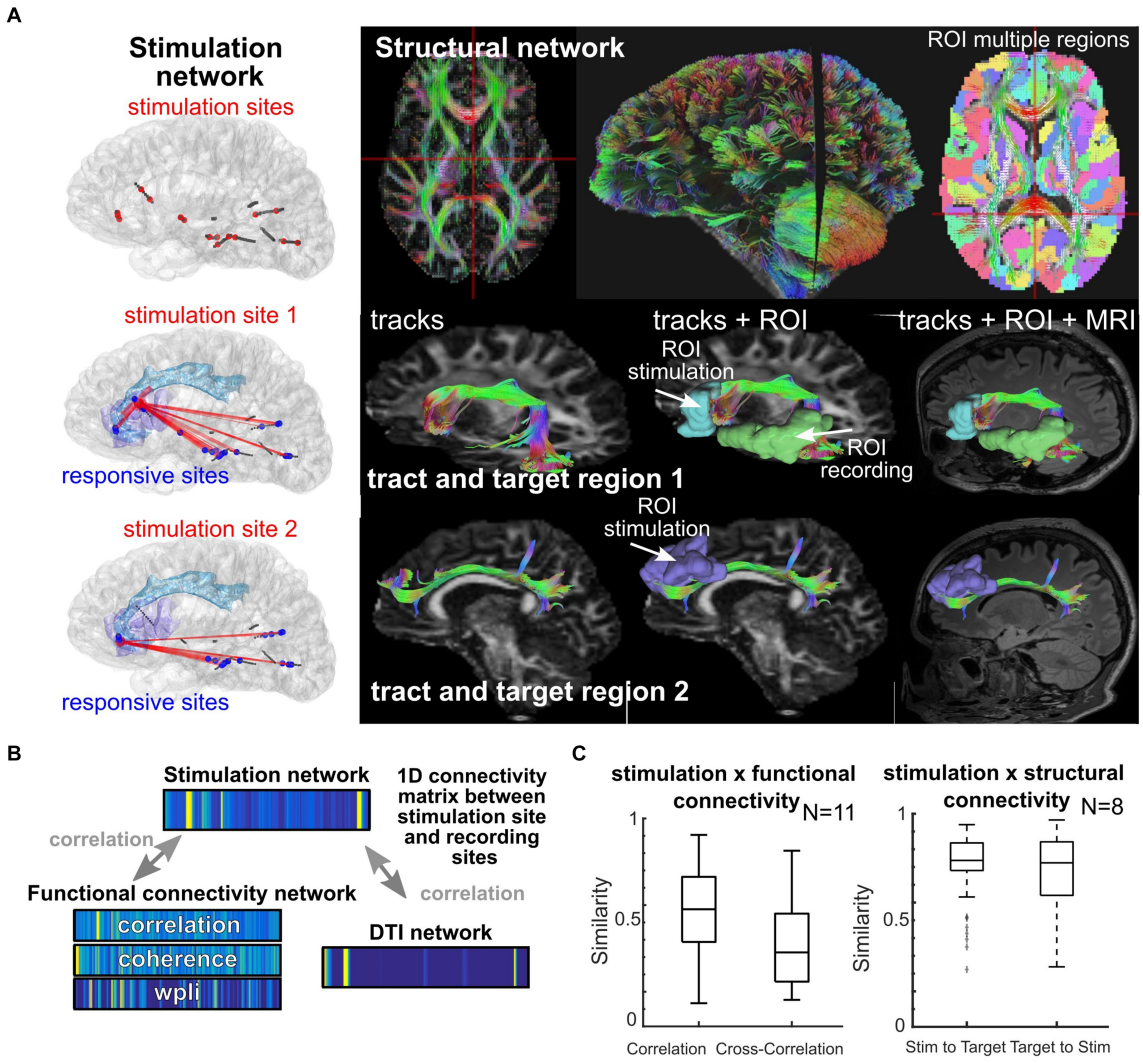
Location and network shapes neural responses. **(A)** Individual participant where stimulation took place (left column) and white matter tracts mapped across the brain for different targets. **(B–C)** Similarity scores calculated between 1D connectivity matrices between stimulation sites and recording sites, with similarity between a given network and the stimulation network (Crocker et al., 2021). The 1D connectivity matrices involve concatenating all connection values between the stimulation site and the recording sites (with a subset shown in **A**), with the connection values either being the strength of the stimulation response from that stimulation site (top), the connectivity measured from the ongoing resting state activity (left bottom 1D matrices), or the DTI connectivity as measured from the DTI weighting from the stimulation and recording ROIs (right bottom 1D matrix).

Personalization of stimulation treatment needs to be framed in the context of brain location. Stimulation location has a substantial impact on the response (Stoney et al., 1968; Brocker and Grill, 2013; Gunalan et al., 2018; Solomon et al., 2018; Trebaul et al., 2018; Anderson et al., 2019; Stiso et al., 2019; Adkinson et al., 2022). Axonal stimulation, for example, is known to increase the spatial spread of neuronal activation beyond the site of stimulation (Nowak and Bullier, 1998a, 1998b; Donos et al., 2016; Horn et al., 2017; Anderson et al., 2019; Stiso et al., 2019; Crocker et al., 2021; Paulk et al., 2022) and is linked to increased therapeutic effectiveness in depression and Parkinson’s disease treatment (Bourne et al., 2012; Horn et al., 2017; Riva-Posse et al., 2018; Crowell et al., 2019; Goodman et al., 2020; Middlebrooks et al., 2021; Scangos et al., 2021a, 2021c, 2021b). Yet, there could be diagnostic and therapeutic reasons to focally target a small grey matter area (cortex) as opposed to stimulating entire networks, such as diagnostically identifying eloquent versus pathologic tissue (Berger and Ojemann, 1992; Borchers et al., 2012; Trébuchon and Chauvel, 2016; Britton, 2018; So and Alwaki, 2018). Functional connectivity reflects ongoing physiological correlations between regions (Bowyer, 2016; Solomon et al., 2018; Hebbink et al., 2019; Crocker et al., 2021) while structural connectivity represents the mapped anatomical connections between regions (e.g., white matter connectivity; Basser et al., 2000; Friston, 2011; Donos et al., 2016; Crocker et al., 2021; Scangos et al., 2021a; Adkinson et al., 2022). We and other groups have found that *stimulation connectivity* maps to both functional connectivity (locally) and structural connectivity (distally; Solomon et al., 2018; Crocker et al., 2021; Scangos et al., 2021a, 2021c; Adkinson et al., 2022; Paulk et al., 2022) and are affected by pathological changes in the networks (Matsumoto et al., 2017; Figure 5). As stimulation connectivity (the spatial spread of stimulation responses) is also determined by the brain region location, we hypothesize that there are consistent, and robust, brain region-specific nonlinear stimulation response maps that operate with separate rules for local (near the stimulation site) and distant (>30 mm from stimulation) recording sites shaped by both functional (local) and structural (distant) connectivity (Figure 5; Crocker et al., 2021). In the case of epilepsy, this connectivity may have possibly been modulated by seizure networks.

To more directly test this interaction of stimulation response, structural connectivity and epilepsy, attempts have been made to better understand the amalgamated structural-functional network interplay, through structural, diffusion, and functional imaging as well as IEEG, between MRI-negative and-positive temporal lobe epilepsy (TLE) among key TLE network nodes which is an excellent example of personalized application of stimulation along the lines of “*How do we get to that Neurostimulationist vision of personalized closed loop therapy?*” The seizure onset network in TLE has been associated with neuronal disorganization on histology, IEEG, structural and diffusion imaging, and it is thought to be due in part to seizure activity and aberrant neuronal connections (Van Paesschen et al., 1997; Frater et al., 2000; Yogarajah and Duncan, 2008; Lemkaddem et al., 2014; Ferrari-Marinho et al., 2015; Gleichgerrcht et al., 2015; Alizadeh et al., 2019). While this interplay between structural neuronal disorganization and the seizure onset was described in TLE-mesial temporal sclerosis (MTS)—particularly in the ipsilateral hippocampus and CA1 subfield—similar findings were not seen in MRI-negative TLE (Shah et al., 2019a, 2019b). Recent studies in TLE have indicated decreased neurite density in the ipsilateral temporal region, both in MRI-negative and MRI-positive TLE (Sone et al., 2018; Winston et al., 2020). Other modalities, such as fMRI and ASL, have shown differences in local and global functional connectivity between MRI-negative and MRI-positive TLE (Vaughan et al., 2016; Lee et al., 2021). PET imaging has also been used to identify seizure onsets in MRI-negative TLE (Gok et al., 2013). The structural and functional networks of MRI-negative and-positive TLE differ by modality, but also differ relative to one another.

### 4.2 Biomarkers + machine learning: neural biomarkers may allow for real-time personalized targets for stimulation

While other clinical markers, like heart rate for vagal nerve stimulation (VNS) devices, and MRI biomarkers, especially for surgical resections, have been widely available for many years, an exciting development in the field of neuromodulation in the past few years has been the identification of clinically relevant neural biomarkers associated with stimulation that can be used to diagnose and treat brain dysfunction. For example, stimulation-induced decreases in gamma (31–150 Hz) oscillations in the amygdala (Scangos et al., 2021a) and increased beta-band (13–30 Hz) oscillations in the subcallosal cingulate (Alagapan et al., 2023) have both correlated and been mapped to the alleviation of depression symptoms. Some devices, like the Medtronic DBS platform, have also relied on tracking local field potentials (LFPs) to provide operators with helpful information to adapt the stimulation parameters (Yang et al., 2023). For instance, in a recent study involving long-term tracking of local field potential (LFP) dynamics with stimulation, researchers demonstrated that stimulation improved independently measured depression scores which mapped to LFP biomarkers in the subgenual cingulate (Veerakumar et al., 2019; Alagapan et al., 2023). Additionally, future clinical applications involving voltametric measurements of neurotransmitters could help produce a better understanding of the underlying mechanisms involved in responses in neurostimulation and provide another source of feedback; in addition, this could be used to elucidate some of the synergistic effects of medications and stimulation (Albert et al., 2009; Van Gompel et al., 2010; Chang et al., 2012; Paek et al., 2013).

This work highlights a direct correlation between intracranial stimulation brain responses, biomarkers of pathological brain dynamics, and therapeutic efficacy in Parkinson’s disease, OCD, epilepsy, and depression (Nair et al., 2020; Sheth et al., 2021; Scangos et al., 2021a, 2021c; Bahners et al., 2022; Alagapan et al., 2023). Clinically relevant biomarkers have taken the form of induced intracranial oscillations in specific frequency bands (Provenza et al., 2019, 2021; Basu et al., 2021; Scangos et al., 2021a), voltage responses to identify connected sites (Miocinovic et al., 2018; Sheth et al., 2021; Scangos et al., 2021c, 2021a), and combinations of LFP features including network connectivity (Alagapan et al., 2023). Interestingly, SPES responses have been mapped to targets involved in mood (where modulation involves trains of stimulation), highlighting the usefulness of different patterns of stimulation in predicting and engaging clinically relevant biomarkers (Adkinson et al., 2022). Therefore, as the field has begun to identify targeted intracranial biomarkers for stimulation efficacy, it becomes essential to both understand and predict stimulation input–output relationships on the level of the physiological signal.

In the field of epilepsy, efforts have also been made to identify response-predictive biomarkers. For instance, invasive and non-invasive connectivity measures have been used to predict the patient’s response to RNS, suggesting that network connectivity can serve as biomarkers of RNS therapeutic effectiveness and identify the patients who may benefit from such treatments (Fan et al., 2022; Scheid et al., 2022). While these studies focus on modulation within the seizure onset area, some studies aimed to predict the effectiveness of neurostimulation within the thalamus (Aiello et al., 2023; Yang et al., 2023). Aiello et al. reported that higher theta power in the anterior nucleus of the thalamus (ANT) recorded during the DBS device implant was associated with better treatment outcomes, although it is not clear whether this benefit could be realized by a reduction in this power (or other changes within the ANT).

We posit that predicting these stimulation parameter combination-biomarker relationships could only truly be bolstered using machine learning (ML) tools. In an answer to the second question of “*How do we get to that Neurostimulationist vision of personalized closed loop therapy?*,” tools provided through mML, a field within artificial intelligence (AI) that helps build algorithms to better understand data and predict potential outputs. While unsupervised machine learning can help us discover some novel biomarkers by better understanding the data, supervised machine learning techniques are typically used to link biomarkers and neurostimulation parameters to clinically relevant findings. Deep learning, a subfield within machine learning, is used especially in some of the complex data sets to help discover complex patterns; the aptly-named neural network models, which mimic the brain by using layers of connected simple functions (like neurons) to process data, serve as a key component of deep learning. ML approaches offer a more general toolkit where multiple neural features can be combined nonlinearly to associate responses with stimulation parameters, unconstrained by previous assumptions of stimulation input–output response oscillatory relationships (Alagapan et al., 2023; Merk et al., 2023). As stimulation parameter space and its relationship to neural activity is indeed complex, ML provides an additional powerful set of tools, particularly to reverse engineer a stimulation response relative to stimulation parameters (Prato and Zanni, 2008; Roy et al., 2019; Hecker et al., 2021; Huang et al., 2022). Recently, considerable advances using ML have related stimulation to neural oscillations (Schmidt et al., 2020), classifying responses to amygdala stimulation (Sendi et al., 2021), identifying stimulation-induced biomarkers of alleviating depression (Alagapan et al., 2023), EEG determination of brain age (Paixao et al., 2020), EEG signatures of consciousness (Sun et al., 2019; Kashkooli et al., 2020; van Sleuwen et al., 2021), sleep (Abou et al., 2020; Sun et al., 2020), and seizures (Brandon Westover et al., 2013; Johansen et al., 2016; Struck et al., 2019), and chronic DBS/RNS recordings to decode seizures, emotion, and movement (Merk et al., 2022, 2023). The powerful combination of ML, biomarkers and stimulation has been highlighted recently with identifying stimulation-related LFP biomarkers best mapped to clinically relevant depression scores on a per-patient basis (Alagapan et al., 2023).

As one answer to “*How do we get to that Neurostimulationist vision of personalized closed loop therapy?*,” we propose the per-patient basis of using ML combined with network connectivity as well as personalized biomarker tracking are all key steps toward enabling optimized and personalized treatment plans. Indeed, if we are able to harness these tools in combination with closed loop algorithms that can adapt to ongoing biomarker changes while being monitored by trained medical providers (Sun and Morrell, 2014; Widge et al., 2017; Basu et al., 2021; Jarosiewicz and Morrell, 2021), we might be that much closer to an onboard treatment system that could give patients freedom from symptoms with reduced need for in-person clinic visits.

In other words, we are at a pivotal and data-rich era of shared intracranial data where clinical decision making could be informed by advanced ML approaches to get us to the original prescriptive Neurostimulationist visit (Figure 1). In addressing the question “*How do we get to that Neurostimulationist vision?*” by laying out a stimulation atlas roadmap, personalized network connectivity, and ML techniques, we could be that much closer to an envisioned robust clinical neuromodulatory treatment.

## 5 How far are we from implementing the neurostimulation visit of the future and tailoring treatment with responsive neurostimulation?

While neurostimulation approaches are currently used in neurologic and psychiatric diseases, the standardization and personalization approaches previously described have yet to be fully implemented in clinical practice, which is one reason we continue to ask the third question of “*How far are we from a Neurostimulationist prescription vision of personalized, closed-loop neurostimulation therapy?*.” However, an encouraging result is that some patients with severe epilepsy have been receiving implanted responsive neurostimulation devices for the last decade with promising results.

### 5.1 Neurology implementation: aiming at a moving target?

As the epilepsy field has moved from a lesional to network approach to intervention, closed-loop, chronic neurostimulation through the NeuroPace responsive neurostimulator (RNS) has become the intervention of choice for some of the most difficult epilepsy cases. However, the current approach to RNS in epilepsy relies primarily on trial and error, which, itself, addresses the third question of “*How far are we from a Neurostimulationist prescription vision of personalized, closed-loop neurostimulation therapy?*” and highlights the need for identifying standardized results, underlying mechanisms, and developing ML models. In the typical workup to be able to implant RNS devices, patients will undergo a battery of tests which include structural MRI, functional MRI, PET scans, and intracranial EEG recordings to capture seizure onsets and spread. More recently, the RNS device has been used to target various nuclei of the thalamus as an alternative mode of therapy, especially in patients with multi-focal or broad epilepsy (Roa et al., 2022; Sisterson et al., 2022; Nanda et al., 2024). The use of thalamic RNS was inspired by the promising result of thalamic DBS trials, in which some patients benefited from thalamic stimulation (Fisher et al., 2010; Li and Cook, 2018; Fisher, 2023). Candidates for a thalamic RNS receive stereoelectroencephalography (sEEG) electrodes within various nuclei of the thalamus to ensure the involvement of the thalamus within the seizure network (Gadot et al., 2022). This is crucial for RNS use since the device sends a stimulating signal upon sensing an appropriate neural signature, such as the onset of a seizure (Sun et al., 2008; Sun and Morrell, 2014). Based on these results, the physicians determine the ideal location to implant the RNS electrodes (within the seizure focus or a specific thalamic nuclei). More frequently, the RNS electrode targets the seizure onset zone directly (very often in the mesial temporal lobes, since temporal lobe epilepsy is among the most common variants).

**Figure 6:**
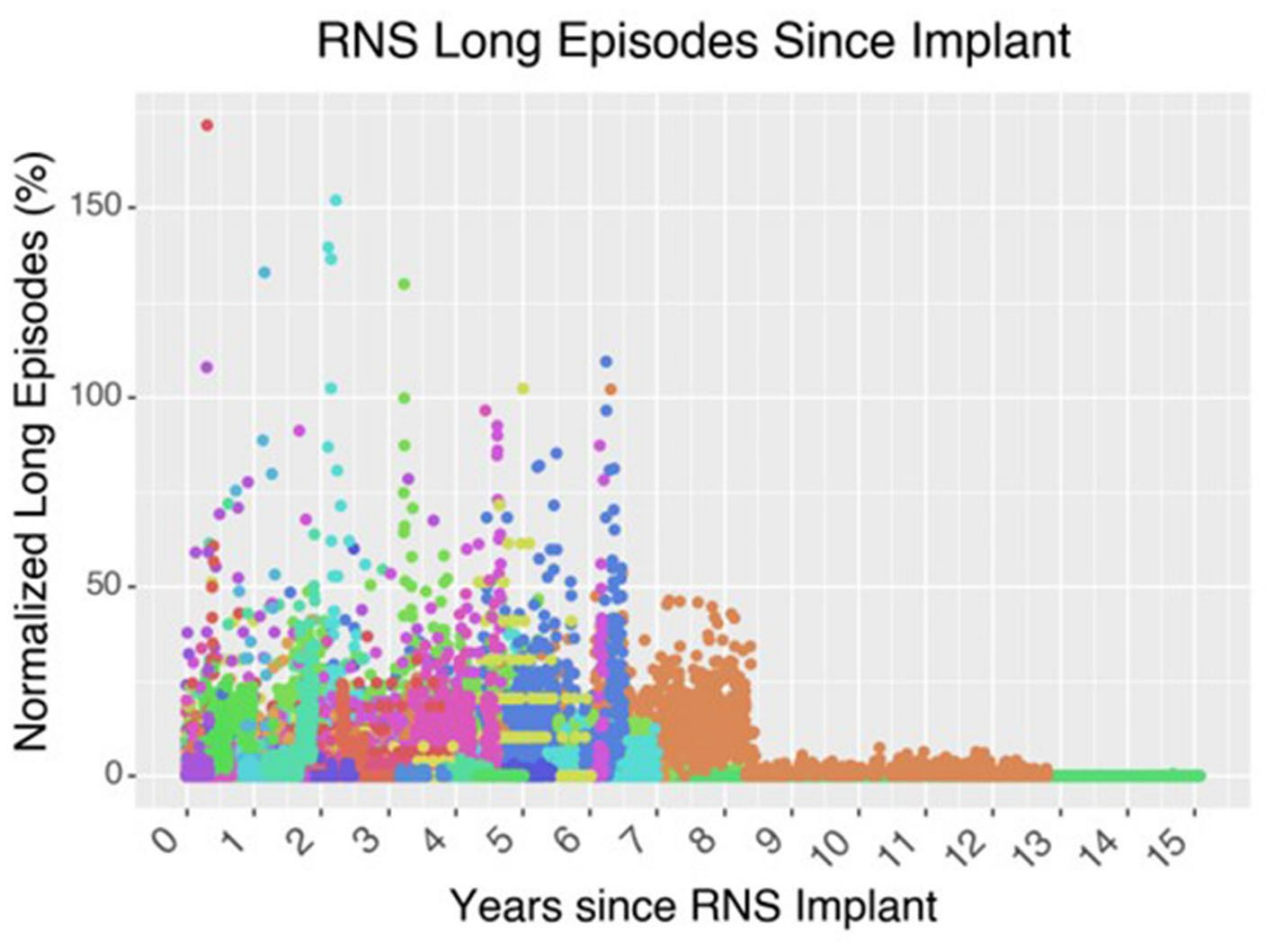
Effect of neurostimulation on seizure control over time. In this graph of 62 patients who underwent RNS implantation at the Massachusetts General Hospital up until 2021, the cohort demonstrates an overall decrease in the number of long episodes per month compared to the initial baseline during the first 3-months after implantation. Long episodes often represent prolonged ictal activity captured on the ECOG recording of the RNS device. Each color is a different patient.

After a patient is implanted with the RNS device, the patient undergoes a several-week period of recording baseline brain activity and seizures; the seizures are registered when the patient swipes a magnet indicating that an event occurred. Over time, the neurologist will work with a specialist from NeuroPace to adapt the detection parameters to capture these events. Once the seizures are thought to be adequately captured by the detection parameters, stimulation is turned on and parameters are modified over several weeks, typically by increasing the charge density. Over subsequent office visits, the neurologist and the NeuroPace specialist will modify the detection and stimulation parameters to try to capture as many seizures as possible and to abort them as well. This entire process can take months for an acceptable response, and for some patients ongoing visits are required, year-after-year, to chase these seizures and improved the responsiveness of the neurostimulation (Figure 6). Some studies aim to reduce the time of achieving favorable outcomes. For example, similar electrographic patterns can be identified between patients who have just received the RNS device and existing RNS patients who have already seen substantial improvement in their seizure control (Arcot Desai et al., 2022). In this case, similar stimulation parameters may be suggested for the new patients, with the goal of reducing the number of visits required to achieve the same favorable response. However, since it now appears that a substantial portion of the benefit arises from long-term epilepsy network reorganization, it may be time to consider a possible better way (Kokkinos et al., 2019; Khambhati et al., 2021; Kundu et al., 2023).

While to date RNS has been used for over a decade, we are only now learning about its true mechanism of action which could involve more long-term network remodeling than short term seizure interruption. Indeed, this point alone highlights why we continue to ask, and are not able to fully answer, “*How far are we from a Neurostimulationist prescription vision of personalized, closed-loop neurostimulation therapy?*” For instance, temporal lobe epilepsy has shown significant improvement with RNS, with some studies showing a 58–70% median seizure reduction, and a major focus of the field has been better tailoring the RNS approach to improve seizure control in a consistent and methodical way (Yang et al., 2022). Although the mechanism of action of RNS was originally thought to be immediate and abortive, based on stopping epileptogenic activity early before it could proceed into a seizure, recent studies have challenged this assumption due to the extensive daily stimulation and gradual response to treatment, which instead points to long-term remodeling of epileptogenic networks (Nair et al., 2020). Recent work indicates that there is a frequency-dependent connectivity change based on intracranial EEG in the first year of RNS that correlated with seizure reduction and support the network reorganization hypothesis (Khambhati et al., 2021). Notably, these epileptogenic networks are not static, and change in response to resection and neurostimulation. Following temporal lobectomy, there are widespread functional connectivity changes, especially in the contralateral hippocampus and thalamus, and can be dependent on the extent of resection (Busby et al., 2019; Morgan et al., 2019). A recent 9-year follow-up study of RNS outcomes shows that over 50% of patients are RNS responders (≥ 50% reduction in seizures) after 1 year, and over 70% respond at 3 years (Nair et al., 2020; Jarosiewicz and Morrell, 2021). Some early RNS studies have shown that increased connectivity to the medial prefrontal and cingulate cortices, among other locations, were associated with better seizure control (Charlebois et al., 2022). Additionally, these connectivity changes are not just be due to interruption of seizure activity; instead, RNS induces chronic network changes that predict better outcomes in TLE (Khambhati et al., 2021). RNS, because of its chronic, closed-loop neurostimulation and electrocorticography (ECOG), has the potential to improve seizure control through a network-based approach and elucidate seizure networks. Not only this, but timing stimulation to periods outside of seizures or timed to periods of sleep could prove to be more beneficial (Anderson et al., 2024; Suresh et al., 2024).

These is most likely the route to address the central question of “*How do we get there?*” By combining the machine learning and network approaches, we may be able to limit the neurostimulation parameter space for our patients to enable improved and more flexible seizure control. Through incorporating a patient’s various imaging scans, intracranial EEG, and *in-vivo* neurostimulation results during intracranial EEG monitoring, we would be able to develop brain maps to help guide the RNS placement, seizure detection parameters, and therapeutic stimulation parameters for every patient. Based on the known long-term network changes associated with a good response to RNS, understanding and treating the patient’s brain and connections in a holistic way becomes the priority as opposed to solely interrupting seizures; various brain states (awake v asleep) and the extent of epileptiform activity present will likely play a larger role in this approach as a result. While there would be some degree of standardization through the limited neurostimulation parameter space, the approaches would be individualized and likely different for every patient; it could be determined that the ideal treatment for a given patient is to implant a region outside the seizure onset zone that can help modulate the network and to target EEG activity that is not necessarily epileptic in nature in order to help control seizures. By standardizing and personalizing neurostimulation treatments in this manner, the hope is that this methodical approach to neurostimulation will lead to consistently good outcomes in epilepsy and other brain diseases and disorders.

## 6 Conclusion

Our perspective piece shows that while we have not yet implemented a standardized and personalized approach to neurostimulation therapy and management, recent advances have removed substantial limitations and brought us much closer to that goal, which we address by asking (1) *Why aren’t we “there” now?* (2) *How do we get there?* and (3) *How far are we away from a Neurostimulationist prescription vision?* Some of the concerns about large parameter spaces of inputs (stimulation), outputs (brain responses), and modulatory factors (awake v. asleep brain states) have been assuaged through mechanistic understandings of stimulation effects and big data approaches; this in turn has enabled a smaller neurostimulation parameter space to be revealed, which can be amenable to standardization. Beyond standardization, another major concerning limitation was accounting for personalization, specifically the differences in individual patient brains. However, this has proven to not be insurmountable, since neurostimulation responses have been shown to be constrained by connectivity and neuroanatomy, and recent machine learning approaches in biomarker analyses have opened the door to a future of personalized neurostimulation maps for individual conditions. Finally, the recent successes in implementing a responsive neurostimulation device for epilepsy treatment and management provide optimism in eventually bringing these advances from the computer desk to the patient’s bedside. As research continues into the standardization, personalization, and clinical implementation of brain neurostimulation, we move closer to a future where brain neurostimulation therapies can be a consistently effective form of treatment for brain diseases and disorders: Just as an orthopedic cast can now reset the bones of a fractured limb with a high degree of accuracy, so too will brain stimulation treatments eventually repair disorders of the brain in a dependable manner.
